# Seasonal reversal in phytoplankton assembly mechanisms: stochastic dominance in autumn vs. deterministic control in spring within the middle and lower reaches of the Yellow River

**DOI:** 10.3389/fmicb.2025.1610438

**Published:** 2025-07-14

**Authors:** Dahai Zeng, Houkuan Ding, Yuanyuan Tang, Yunni Gao, Jialin Jin, Xiaofei Gao, Jingxiao Zhang, Huatao Yuan, Jing Dong, Xuejun Li

**Affiliations:** ^1^College of Fisheries, Henan Normal University, Xinxiang, China; ^2^Observation and Research Station on Water Ecosystem in Danjiangkou Reservoir of Henan Province, Nanyang, China; ^3^The National Ecological Quality Comprehensive Monitoring Station (Hebi Station), Hebi, China

**Keywords:** the Yellow River, phytoplankton, community assembly, autumn, spring

## Abstract

Phytoplankton communities play a crucial role in riverine ecosystems, yet their assembly mechanisms in high-silt environments remain poorly understood. This study investigated seasonal variations in phytoplankton community structure and assembly mechanisms in a riverine environment with high silt loads. Phytoplankton and physicochemical water quality factors were analyzed in the middle and lower reaches of the Yellow River during two seasons: November 2023 (autumn) and April 2024 (spring). A total of 110 algal species from seven phyla were identified, with Chlorophyta being predominant in species richness and Cyanophyta in cell density. Both species richness and abundance were lower in autumn than in spring. The α-diversity analysis revealed that the Pielou’s eveness index was significantly higher in spring compared to autumn. Non-metric multidimensional scaling (NMDS) based on Bray–Curtis distances showed significant seasonal differences in phytoplankton community composition. Furthermore, β-diversity decomposition analysis revealed that turnover was the dominant component in both seasons, but the proportion of nestedness was significantly higher in spring compared to autumn (*p* < 0.05). Based on the analyses of the dispersal–niche continuum index (DNCI) and the modified stochasticity ratio (MST), this study demonstrates pronounced seasonal variations in the assembly mechanisms governing phytoplankton communities in the middle and lower reaches of the Yellow River. In autumn, stochastic processes, primarily driven by dispersal, accounting for 58.85% of the community assembly. In contrast, deterministic processes, largely shaped by niche selection, contributing 65.05% to the community assembly in spring. The community structure of phytoplankton in this region is shaped by the combined effects of geographical factors, elevation, and environmental variables, with particularly pronounced seasonal variations in environmental drivers—total nitrogen (TN) emerges as the primary factor influencing autumn community assembly, while spring community structure is mainly regulated by silica (SiO₂) and pH. This study deepens the understanding of phytoplankton assembly mechanisms in sediment-rich rivers and provides fundamental data for phytoplankton construction mechanisms and aquatic biodiversity conservation in the middle and lower reaches of the Yellow River.

## 1 Introduction

As primary producers in aquatic ecosystems, the mechanisms governing phytoplankton community assembly directly influence material cycling, energy flow, and functional stability within these ecosystems (Cardinale et al., 2002). An in-depth analysis of the assembly processes of phytoplankton communities not only enhances our understanding of the mechanisms governing biodiversity formation and maintenance but also offers a theoretical foundation for predicting ecosystem responses to environmental changes (Wu et al., 2023). Community ecology research indicates that the formation of phytoplankton communities is influenced by both deterministic and stochastic processes (Vellend, 2010). The deterministic process is primarily driven by environmental filtering, wherein species that are well-adapted to specific conditions are selected through mechanisms such as resource competition, predation pressure, and environmental selection. Conversely, stochastic processes shape community structure through diffusion limitation, ecological drift, and random extinction events, typically prevailing when environmental selection pressures are weak or when conditions become homogenized (Isabwe et al., 2018). Recent advancements in community ecology theory and analytical methodologies have highlighted that deterministic and stochastic processes are not mutually exclusive but instead coexist, dynamically shaping community structure in a state of equilibrium (Ning et al., 2020). The analysis of this dynamic equilibrium holds significant implications for understanding ecosystem stability and resilience, particularly in the context of global climate change and escalating anthropogenic activities.

In terms of analytical methodologies, researchers have developed various models and indices to elucidate the mechanisms underlying community assembly. To quantify the relative contributions of deterministic and stochastic processes, the dispersal–niche continuum index (DNCI) proposed by Vilmi et al. (2021) represents a significant advancement in the traditional understanding of community assembly at the α-level, providing a new quantitative tool for studying β-level community assembly processes and enabling researchers to evaluate community assembly mechanisms across different spatial scales. Brown et al. (2022) used the DNCI method to discover that random processes dominated the construction of rotifer communities in the shallow water area of temporary drought. Additionally, Ning et al. (2020) developed a mathematical framework to quantify ecological stochasticity across environments by creating the modified stochasticity ratio (MST). Chao et al. (2025) used the MST method to prove that the phytoplankton community process in plateau rivers is dominated by randomness.

The Yellow River, recognized as the most silt-laden river in the world, spans approximately 5,464 km and traverses several geographic regions, including the Tibetan Plateau, Loess Plateau, and North China Plain, resulting in a complex hydrological and ecological environment (Song et al., 2020). Several existing studies have been conducted in the field of phytoplankton ecology within the Yellow River Basin (Ding et al., 2022; Han et al., 2024; Song et al., 2020; Yi et al., 2024). However, there is still conflicting evidence regarding the driving factors of phytoplankton community composition and assembly mechanisms. The variation partitioning analysis showed that the contribution of pure spatial factors (geographic distance) to the succession of the phytoplankton community structure along the Yellow River was four times higher than that of the combined effects of water quality and climate conditions (Ding et al., 2022). Another study based on algal community across different habitats revealed that sediment concentrations was a critical factor to influence algal community assembly, with homogeneous selection exerting a more prominent influence in habitats rich in sediment, i.e., the middle and lower reaches (Han et al., 2024).

According to the Yellow River Conservancy Commission’s Silt Bulletin (2019–2023), the annual average silt discharge at key hydrological stations in the source and upper reaches over the past 5 years has been 35 million tons, while in the middle and lower reaches, it has been 219 million tons, resulting in a difference of 184 million tons (Supplementary Figure S1). To reduce sediment deposition in the middle and lower reaches, the water-sediment regulation scheme of the Xiaolangdi Dam, has been implemented annually during the summer months since 2002 (Song et al., 2020). Hence, the water and sediment discharge caused a marked changes of hydrodynamic conditions during summer (Zhao et al., 2022). The different hydrodynamic conditions and water quality may lead to seasonal changes of phytoplankton assembly process. A recent study found dispersal limitation was more influential on phytoplankton community assembly in summer and ecological niche differentiation was more dominant in spring (Yi et al., 2024). However, it lacks data on autumn, which is the key transitional stage for the reconstruction of phytoplankton communities after water-sediment regulation scheme. Multiple studies have shown that environmental changes in spring and autumn have the most significant impact on the structure of phytoplankton communities (Fu et al., 2024; Xu L. et al., 2023; Xu S. et al., 2023). The research background indicates that there are significant seasonal differences in environmental factors (flow velocity, water quality, organic matter and nutrient salt concentration, etc.) of different sections of the Yellow River in spring (dry season) and autumn (wet season) (Ni et al., 2023; Dou et al., 2022; Zhao et al., 2018; Hu et al., 2023; Liu et al., 2025). Based on this, this study proposes a scientific hypothesis: the construction mechanism of phytoplankton communities in the middle and lower reaches of the Yellow River shows significant seasonal differences between autumn and spring.

Hence, to further investigate the seasonal assembly mechanisms of phytoplankton communities, particularly in the middle and lower reaches of the Yellow River, this study integrated geographic factors, elevation, and environmental variables, using analytical methods such as DNCI and MST to qualitatively and quantitatively evaluate the roles of deterministic and stochastic processes in the assembly of phytoplankton communities during spring and autumn.

## 2 Materials and methods

### 2.1 Study area

**Figure 1:**
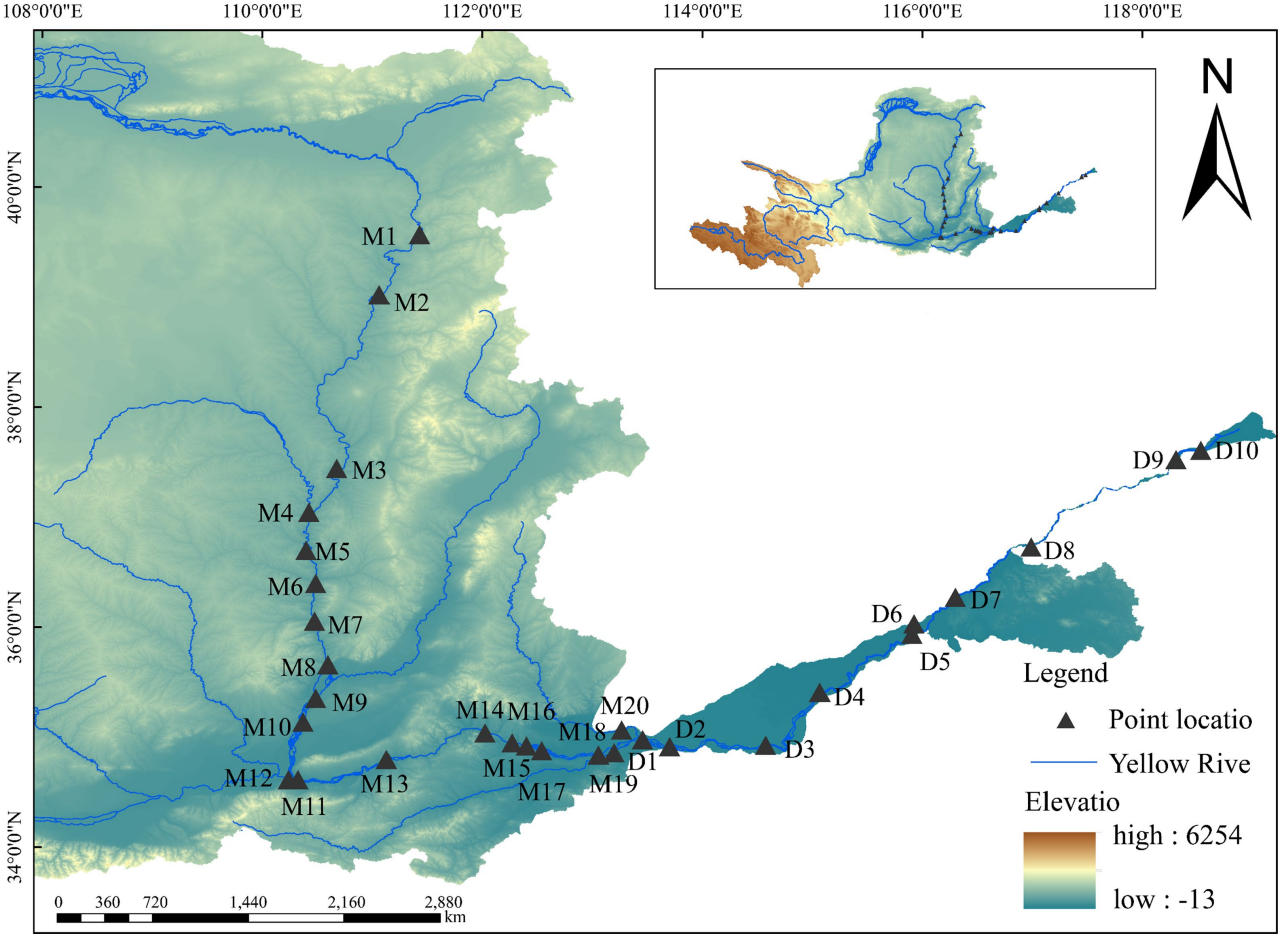
Map of sampling sites in the study area.

The middle and lower reaches of the Yellow River, extending for 2,064 km from Tuoketuo County in Inner Mongolia to Dongying City in Shandong Province, traverse two distinct geographic regions: the Loess Plateau and the North China Plain. This segment of the river exhibits unique hydrological and geomorphological characteristics and is recognized as the most silt-laden river globally (Ma and Huang, 2016). The topography in this area transitions gradually from the canyon landscapes of the Loess Plateau to the flat alluvial plains, with an elevation differential of up to 904 metres. This significant topographical variation exerts a considerable influence on the river’s hydrodynamic characteristics and silt transport processes (Figure 1). The Loess Plateau, characterized by sparse vegetation and a loose soil structure, coupled with recurrent heavy rainfall and substantial soil erosion, results in the middle reach of the Yellow River being one of the most silt-laden river segments worldwide. Statistical analysis reveals that the annual silt discharge in the middle reaches of the Yellow River is approximately 1.6 billion tons, accounting for over 90% of the river’s total silt discharge (Xu et al., 2022). As the river progresses into the lower reaches, the channel slope diminishes sharply, leading to a reduction in flow velocity and an increase in silt deposition. This phenomenon is termed the “suspended river” (Xu L. et al., 2023; Xu S. et al., 2023). This distinctive hydrological and geomorphological characteristic is responsible for the annual elevation of the riverbed in the lower reaches, as well as a substantial increase in turbidity and silt concentration in the water, thereby intensifying the ecological complexity of the middle and lower reaches.

We established 30 sampling sites in the middle and lower reaches of the Yellow River (Figure 1), following the Technical Guidelines for Aquatic Ecological Monitoring: Monitoring and Evaluation of Riverine Aquatic Organisms for sampling site design (Ministry of Ecology and Environment of the People’s Republic of China, 2023). Specifically, 20 sites were located in the middle reaches (M1–M20) and 10 in the lower reaches (D1–D10). Most sites were situated along the mainstem of the river, with exceptions including M9, M11, M19, D1, and D6, which were positioned at the confluences of the Yellow River’s mainstem and its major tributaries (Supplementary Table S1). Surveys were conducted in November 2023 (autumn) and April 2024 (spring).

### 2.2 Sample collection and environmental factor measurement

The collection and identification of phytoplankton samples adhered to the standard protocols outlined in the “Methods for the Study of Freshwater Phytoplankton” (Zhang and Huang, 1991). Qualitative samples were collected using a No. 25 plankton net (mesh size 64 μm) employing an “∞” motion at a depth of 0.5 m. Following 5 min of filtering, the sample was vertically aspirated and immediately preserved in a 4% formaldehyde solution. Upon arrival at the laboratory, species identification was conducted using a LEICA DM500 research-grade light microscope. For quantitative analysis, 1 L of water was collected at the same depth, fixed immediately with Lugol’s solution, and allowed to settle for 48 h. The concentrated sample was then adjusted to a final volume of 50 mL and enumerated under a microscope at 10 × 40 magnification, with each sample counted in triplicate. Diatom samples were treated with hydrogen peroxide for slide preparation and identified using a 10 × 100 oil immersion objective.

An on-site multiparameter water quality analyzer (Pro Quatro, YSI) was employed to measure dissolved oxygen (DO), water temperature (WT). A portable turbidity meter (HACH 2100Q) was utilized for turbidity measurement, while a portable flow velocity (FV) meter (GLOBALWATER, FP111) was employed to determine flow velocity. A global positioning system (GPS) was used to record the longitude, latitude, and elevation of the sampling sites. Nutrient samples, including total nitrogen (TN), total phosphorus (TP), ammonia nitrogen (NH_3_-N), phosphate (PO_4_^3^-P), silicate (SiO_2_), nitrate (NO_3_-N), and chlorophyll a (Chl-a), were transported frozen to the laboratory for analysis, following the procedures outlined in the “Water and Wastewater Monitoring and Analysis Methods” (4th edition) (Wei, 2002). The Analytik Jena multi N/C 3100 (Germany) is used for Total Organic Carbon (TOC) measurement.

### 2.3 Data processing and analysis

The study area map was generated using ArcGIS 10.8. Phytoplankton α-diversity indices were calculated employing the Shannon-Wiener index (Shannon et al., 1950), Pielou’s eveness index (Pielou, 1966), and Margalef’s richness index (Margalef, 1969). The above index calculation was completed in R 4.4.0 using a vegan package. The computation of phytoplankton β-diversity utilized the Baselga framework for β-diversity decomposition, as implemented within the R 4.4.0 package Baselga. The overall β-diversity between spring and autumn sampling sites was determined by calculating the Jaccard similarity coefficient and decomposing it into turnover and nestedness components (Baselga, 2010). In R 4.4.0, the vegan package was employed to perform a non-metric multidimensional scaling (NMDS) analysis based on the Bray–Curtis (BC) distance, with the aim of evaluating disparities in phytoplankton community structure between the spring and autumn seasons. Subsequently, the relationship between the BC distance of phytoplankton communities and geographic, elevation and environmental distances, was assessed to identify decay patterns and elucidate the influence of spatial and environmental factors on community dissimilarity (Hanson et al., 2012). The DNCI index was calculated in R 4.4.0 using the DNCImper software package to determine the main factors influencing community aggregation in each season (Vilmi et al., 2021). The modified stochasticity ratio (MST) was calculated using the NST package in R 4.4.0 to evaluate the relative importance of random and deterministic processes for the construction of phytoplankton communities (Ning et al., 2020). For the corrected random rate analysis, MST values less than and greater than 0.5, respectively, indicate that deterministic and random processes are dominant. Subsequently, the Hellinger transform was performed on the biological data using the R vegan package in R 4.4.0, and then the collinearity analysis of environmental factors was conducted. Variables exhibiting a variance inflation factor (VIF) exceeding 10 were eliminated. Detrended correspondence analysis (DCA) was conducted on the dominant species of phytoplankton using Canoco 5. If the maximum gradient length of each axis exceeded 4, it belonged to a unimodal model and canonical correspondence analysis (CCA) was used. If it was less than 3, it belonged to a linear model and redundancy analysis (RDA) was used. Significance testing, plotting, and data organization for this study were conducted using Origin 2021, GraphPad 8.0.2, and Excel 2022.

The distance-based Moran eigenvector maps (dbMEM) analysis method was utilized to construct the spatial eigenvector variables for the study area (Borcard et al., 2018). The dbMEM analysis was conducted using the dbmem function from the adespatial package in R 4.4.0, resulting in the generation of four spatial variables: MEM1, MEM2, MEM3, and MEM4. These spatial variables were subsequently employed in the following RDA.

## 3 Results

### 3.1 Species composition and diversity

**Figure 2:**
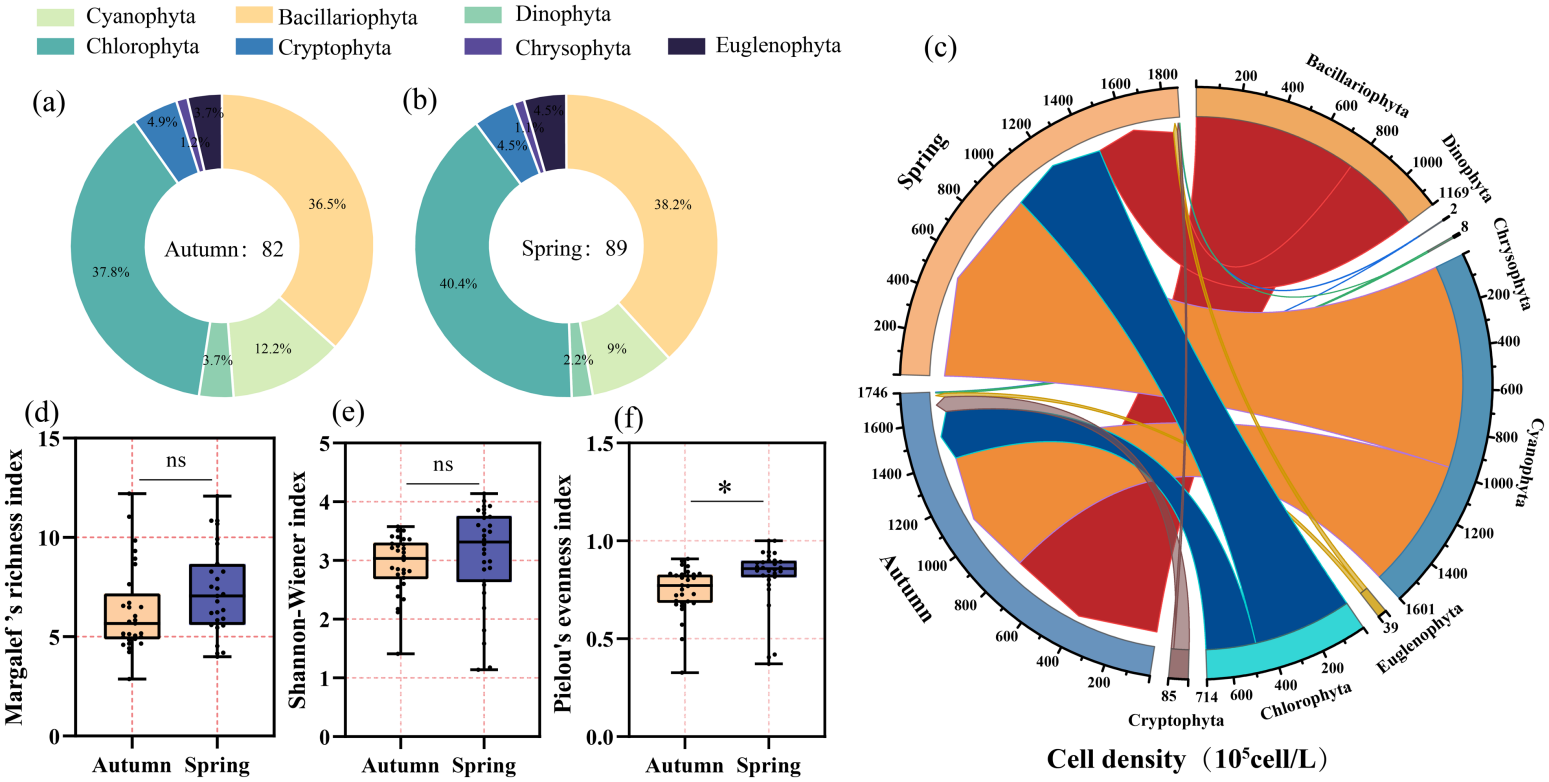
Species richness of phytoplankton in autumn **(a)** and spring **(b)**, cell density of phytoplankton at phylum levels in autumn and spring **(c)** and α-diversity indices: Margalef’s richness index **(d)**, Shannon-Wiener index **(e)** and Pielou’s evenness index **(f)** in autumn and spring.

In the middle and lower reaches of the Yellow River, a total of seven phyla, 69 genera, and 110 species of phytoplankton were identified during the two sampling periods. Chlorophyta was the dominant group, comprising 43 species (39.09%), followed by Bacillariophyta with 39 species (35.45%). Cyanophyta were represented by 13 species (11.81%), while Cryptophyta included five species (5.54%). Dinophyta and Euglenophyta each accounted for 3.63% with four species each, and Chrysophyta had the fewest species, totaling 2 and accounting for 1.81%. In spring, seven phyla, 52 genera, and 89 species were recorded, whereas in autumn, seven phyla, 57 genera, and 82 species were identified (Figures 2a,b).

The total cell density of phytoplankton in autumn was 2.54–759 × 10^5^ cells/L, with the lowest density at site D8 and the highest at site M9. The average density across all sites was 58.3 × 10^5^ cells/L. In spring, the total density was 0.26–1114.75 × 10^5^ cells/L, with the lowest density at site M18 and the highest at site D6, yielding an average density of 62.39 × 10^5^ cells/L. In terms of relative abundance at the phylum level, Bacillariophyta (44.89%) and Cyanophyta (35.69%) dominated in autumn, followed by Chlorophyta (13.72%). In spring, Cyanophyta (52.19%) were the most dominant, followed by Chlorophyta (25.4%) and Bacillariophyta (20.56%) (Figure 2c).

**Figure 3:**
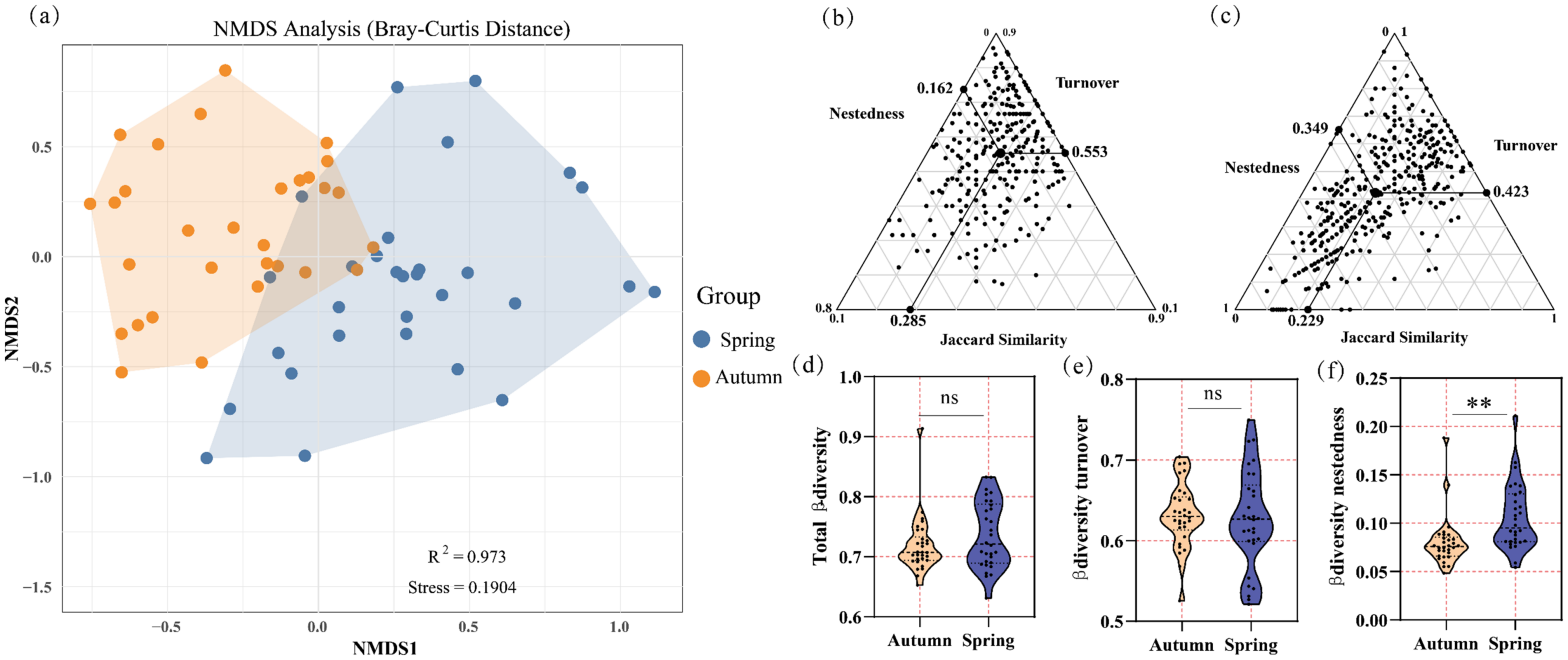
β-diversity of phytoplankton community in autumn and spring. NMDS analysis **(a)**, β-diversity decomposition in autumn **(b)** and spring **(c)**, seasonal differences in total β-diversity **(d)**, in turnover component **(e)** and in nestedness component **(f)**.

An α-diversity analysis of the phytoplankton communities in the middle and lower reaches of the Yellow River revealed no significant differences between the two seasons for both the Shannon-Wiener index and the Margalef’s richness index (*p* > 0.05). However, the Pielou’s evenness index exhibited a significantly higher value in spring compared to autumn (*p* < 0.05) (Figures 2d–f). NMDS analysis of the phytoplankton communities, based on BC distance, indicated significant seasonal differences in community composition (*R*^2^ = 0.973, Stress = 0.1904) (Figure 3a). In autumn, the mean values of β-diversity and its turnover and nestedness components for the phytoplankton community were 0.285, 0.553, and 0.162, respectively (Figure 3b). In spring, the corresponding values for β-diversity and its turnover and nestedness components were 0.229, 0.423, and 0.349 (Figure 3c). The analysis revealed that turnover components dominated in both seasons, with seasonal differences primarily stemming from the nestedness fraction.

### 3.2 Mechanisms of phytoplankton community assembly

**Figure 4:**
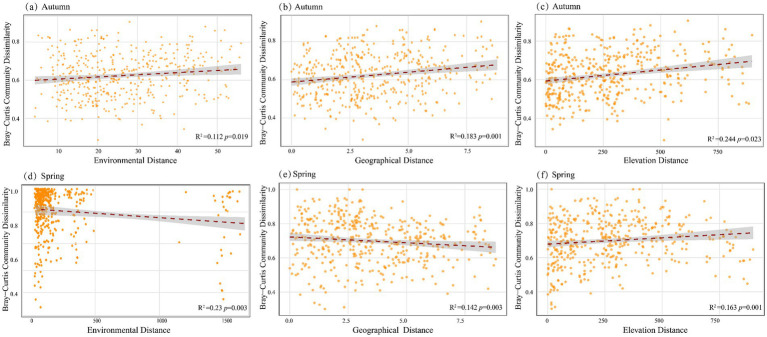
The correlation between BC distance and environmental distance, geographical distance and elevation distance of phytoplankton community in autumn **(a–c)** and spring **(d–f)**.

Correlation analysis using Bray–Curtis distance of phytoplankton communities and environmental, geographic, and elevation distances revealed that the dissimilarity of phytoplankton communities in the middle and lower reaches of the Yellow River during autumn significantly increased with environmental distance (*p* < 0.05), geographic distance (*p* < 0.001), and elevation distance (*p* < 0.05). This indicates a clear pattern of community decay with environmental, geographic, and elevation distances (Figures 4a–c). In spring, however, the dissimilarity of phytoplankton communities significantly decreased with environmental (*p* < 0.05) and geographic distances (*p* < 0.05), yet increased with elevation distance (*p* < 0.001, Figures 4d–f). These findings collectively suggest that phytoplankton community assembly in both seasons is jointly driven by a combination of environmental filtering, geographic dispersal constraints, and elevation heterogeneity.

**Figure 5:**
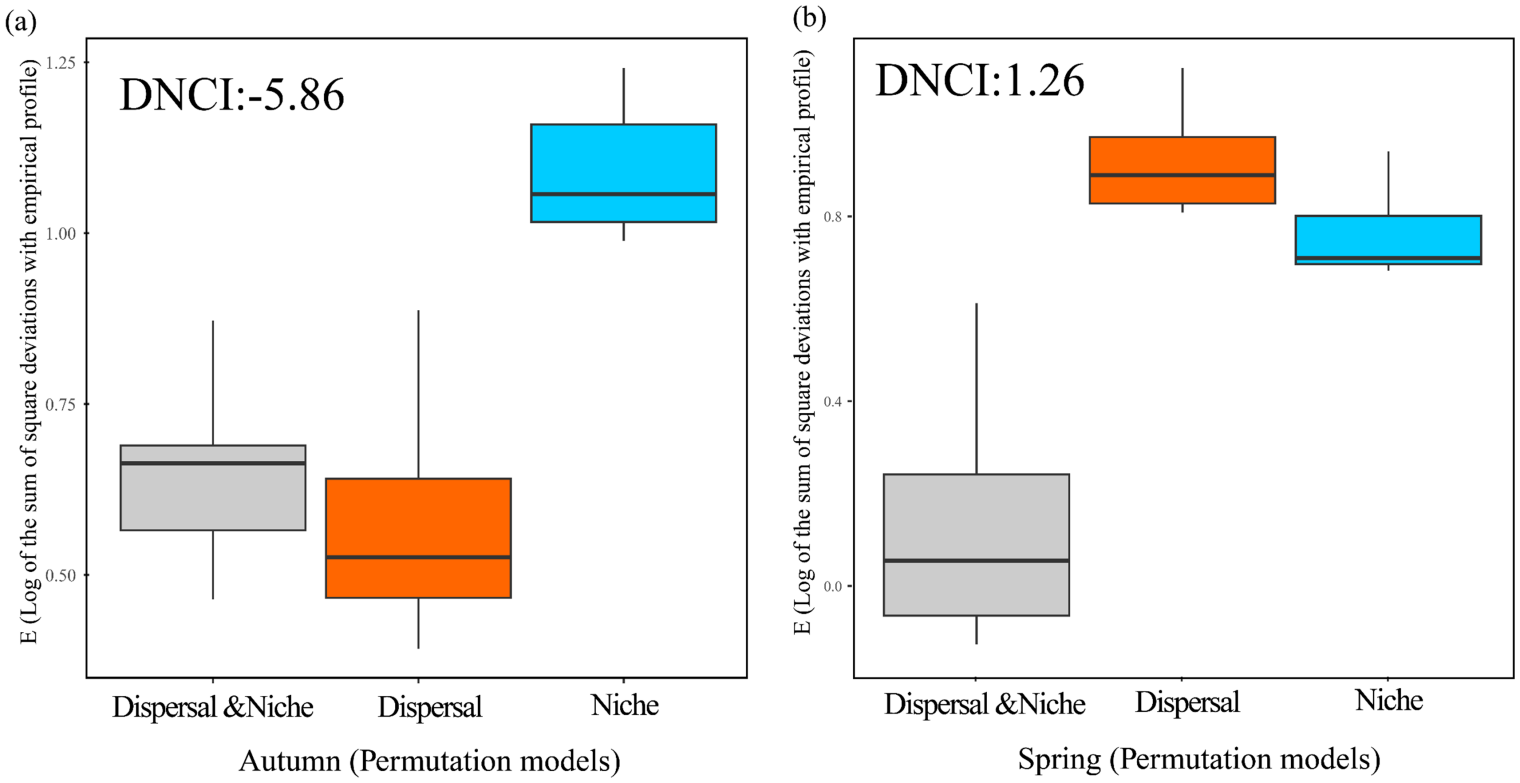
The relative importance of dispersal versus niche processes in driving phytoplankton community assembly in autumn **(a)** and spring **(b)** according to the assessment by PER-SIMPER and the dispersal–niche continuum index (DNCI).

The absolute value of the DNCI index reflects the intensity of community assembly processes and the environmental differences between communities. A negative value indicates that diffusion dominates the process, a value close to 0 suggests that both niche and diffusion processes jointly determine community assembly, while a positive value indicates that niche processes are the primary driving force in community assembly. In this study, PER-SIMPER analysis revealed that in both seasons, the processes governed by niche and diffusion jointly shaped the phytoplankton community, as the *E*-values (logarithm of the sum of squared deviations from the empirical profile) of the “niche + diffusion” process were relatively low. The results indicated that in autumn, the DNCI value was −5.86, suggesting that diffusion was the dominant process, while in spring, the DNCI value was 1.26, indicating that niche processes were the predominant force (Figures 5a,b).

**Figure 6:**
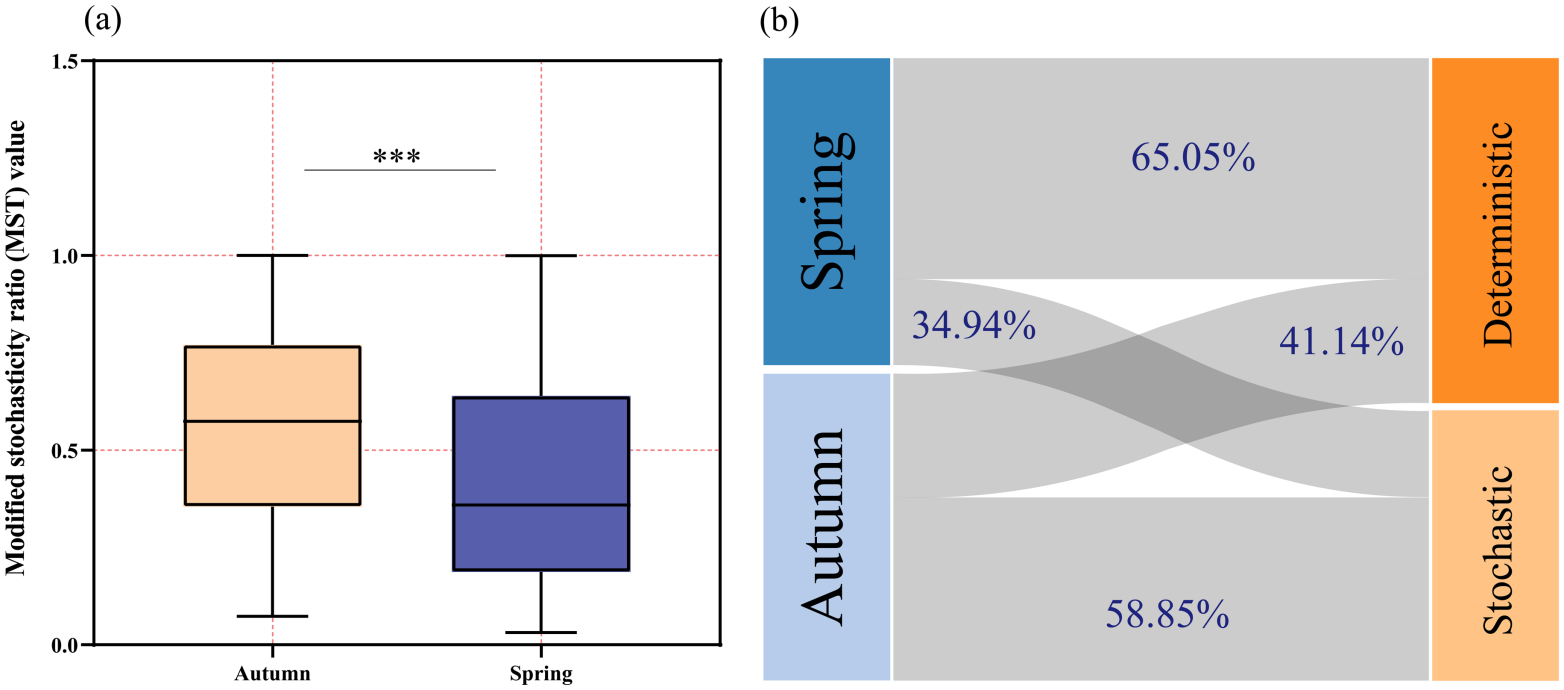
MST-corrected stochasticity ratio values **(a)** and the percentage of stochastic and deterministic processes **(b)** based on the corrected stochasticity ratios.

To elucidate the relative importance of deterministic and stochastic processes in the assembly of phytoplankton communities in the high-silt region of the Yellow River, the modified stochasticity ratio (MST) was employed to quantitatively assess the stochasticity in ecological processes. An MST value greater than 0.5 indicates dominance of stochastic processes, whereas a value less than 0.5 signifies dominance of deterministic processes. Statistical analysis of the MST values greater than 0.5 for the spring and autumn sampling sites revealed that in autumn, stochastic processes accounted for 58.85%, while deterministic processes accounted for 41.14%. In contrast, in spring, stochastic processes comprised 34.94%, and deterministic processes accounted for 65.05% (Figure 6). The analysis of DNCI results in conjunction with MST results suggests that the predominance of stochasticity in autumn phytoplankton communities is attributable to diffusion-driven processes, whereas the dominance of determinism in spring is driven by niche processes. Overall, stochasticity and determinism influence phytoplankton community assembly in the middle and lower reaches of the Yellow River to varying degrees, exhibiting clear seasonal variations.

### 3.3 Spatial and environmental influences on phytoplankton communities

**Figure 7:**
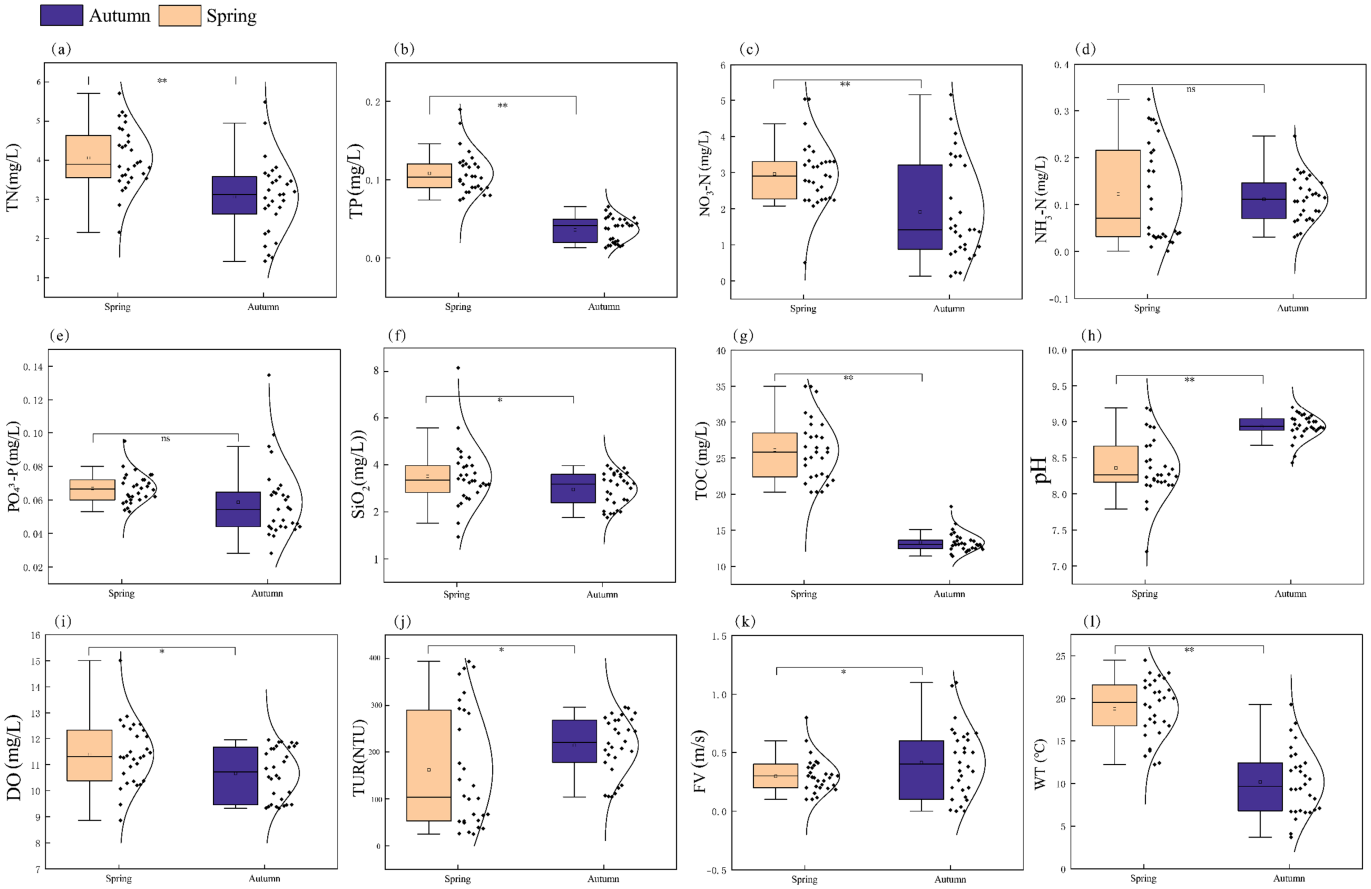
Seasonal variations of physicochemical water parameters in the middle and lower reaches of the Yellow River: total nitrogen-TN **(a)**, total phosphorus-TP **(b)**, nitrate-NO₃-N **(c)**, ammonia nitrogen-NH₃-N **(d)**, phosphate-PO₄3-P **(e)**, silicate-SiO₂ **(f)**, total organic carbon TOC **(g)**, pH **(h)**, DO **(i)**, turbidity-TUR **(j)**, flow velocity -FV **(k)**, and water temperature-WT **(l)**. Significance levels: ^*^p < 0.05, ^**^p < 0.01, and ^***^p < 0.001; ns (not significant).

Comparative analysis of physicochemical parameters between spring and autumn revealed significant seasonal variations in multiple environmental factors (Figure 7). Statistical analysis demonstrated that six parameters, including TOC, pH, TN, NO_3_-N, WT, and TP, exhibited highly significant differences (*p* < 0.01). Four parameters, namely SiO_2_, FV, DO, and TUR, showed significant differences (*p* < 0.05), while PO_4_^3^-P and NH_3_-N displayed no significant seasonal variations (*p* > 0.05). Further analysis revealed that nutrient levels were significantly higher in spring than in autumn (*p* < 0.05), whereas flow velocity and turbidity were significantly higher in autumn than in spring (*p* < 0.05), reflecting the characteristic changes in hydrological conditions across different seasons.

**Figure 8:**
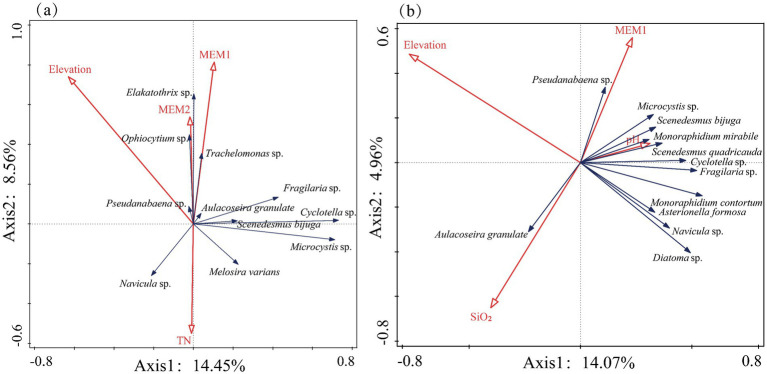
RDA ordination plots showing the relationship between dominant phytoplankton species and key environmental variables in **(a)** autumn and **(b)** spring.

The DCA results for dominant phytoplankton species density in autumn, in relation to environmental, geographical, and elevation factors, revealed a first axis length of 2.5, indicating that RDA could more accurately assess the driving factors of phytoplankton community structure changes. The dominant species are shown in Supplementary Table S2. Based on the VIF values of the explanatory variables and forward selection, the final RDA results identified three significant influencing factors (Monte Carlo test, *p* < 0.05) that explained the changes in phytoplankton community structure: elevation, TN, and the spatial factor MEM1, MEM2. These factors accounted for 23.01% of the variation in phytoplankton along the first two axes, with *p*-values of 0.02, 0.03, and 0.03, respectively (Figure 8a).

For spring, the DCA results indicated a first axis length of 1.2, and the final RDA analysis identified three environmental factors: pH, spatial factor MEM1, and SiO_2_. The *p*-values for these factors were 0.03, 0.04, and 0.03, respectively, with the cumulative explained variation for the first two axes being 19.03% (Figure 8b).

## 4 Discussion

### 4.1 Diversity patterns of phytoplankton communities

The present study identified seven phyla, 69 genera, and 110 species of phytoplankton, predominantly comprising Bacillariophyta, Chlorophyta, and Cyanophyta. They are also the dominant phyla in the Yellow River (Lv et al., 2024). The Shannon-Wiener index and Margalef’s richness index were found to be slightly higher in spring compared to autumn. However, no significant differences were observed. The Shannon-Wiener index and Margalef’s richness index of phytoplankton in the Yarlung Tsangpo River also showed no substantial discrepancies between spring and autumn (Chao et al., 2025). Nevertheless, our study further elucidated a significant disparity in evenness between the two seasons. Significant variations in evenness during spring and summer was also observed in the Yellow River previously (Yi et al., 2024). This discrepancy may be attributed to the distinct environmental factors present in spring and autumn (Lv et al., 2024). In this survey, significant differences were observed in environmental factors, including TOC, pH, SiO₂, NO₃-N, FV, DO, TUR, TN, TP, and WT, between the two seasons (Figure 7).

β-diversity is defined as the variation in species composition among different communities and reflects two distinct ecological processes: turnover (species replacement within communities) and nestedness (the loss or gain of species among communities) (Si et al., 2017). The turnover component accounted for a greater proportion of β-diversity than the nestedness component in both seasons of the middle and lower reaches of the Yellow River. This finding indicates that the differences in species composition primarily arise from spatial species replacement. This is consistent with the findings on the β-diversity of aquatic microbial communities in the Yarlung Tsangpo River Basin and the Liuxi River (Chao et al., 2025; Tan et al., 2023). The higher proportion of turnover components observed in autumn phytoplankton communities is associated with enhanced river diffusion (Chao et al., 2025). A significant enhancement of silt concentrations (TUR, Figure 7j) and flow rates (FV, Figure 7k) was observed in autumn compared to spring in this survey. Hydrodynamics is considered a critical factor shaping the composition of phytoplankton communities in rivers (Zheng et al., 2024). Conversely, the reduced flow, elevated nutrient levels, and increased temperatures may collectively account for the decline in the turnover component observed during the spring season. This leads to greater species overlap among communities and a relatively higher nestedness component in spring. These results are consistent with the prevailing academic consensus that in nutrient-rich environments, phytoplankton community assembly is predominantly governed by niche-based processes (Jiang et al., 2023; Li et al., 2024).

### 4.2 Phytoplankton community assembly mechanisms

The NCM analysis revealed that stochastic assembly was the predominant force shaping Bacillariophyta communities across the entire Yellow River, encompassing 26 river sites and 18 reservoir sites during spring and autumn (Ding et al., 2022). Variance partitioning analysis (VPA) and iCAMP analysis further indicated that homogeneous selection contributed more than 50% to the assembly of both free-living and particle-attached algal communities in the middle and lower reaches of the Yellow River (Han et al., 2024). These findings were derived from 18S and 23S amplicon sequencing data, respectively, highlighting the habitat- and region-dependent variation in algal community assembly processes. The present study elucidates pronounced seasonal variations in the mechanisms governing phytoplankton community assembly in the middle and lower reaches of the Yellow River through the integration of DNCI and MST analyses. In conjunction with another study on phytoplankton community assembly in typical reservoirs and river sections of the entire Yellow River during spring 2024 and autumn 2023, we conclude that stochastic processes, primarily driven by dispersal, dominate phytoplankton community assembly in autumn, whereas deterministic processes prevail in spring in the middle and lower reaches of the Yellow River.

Previous studies suggested that deterministic processes dominate plankton community assembly in lentic aquatic ecosystems such as isolated lakes and reservoirs, while stochastic processes exhibit greater influence on assembly in lotic ecosystems such as interconnected aquatic ecosystems (Yang et al., 2025). The modified stochasticity ratio (MST) indicated that deterministic processes were the predominant factors governing the assembly processes of eukaryotic plankton communities in Liujiaxia Reservoir in upper Yellow River (Chen et al., 2024). However, the community formation pattern of 5 drinking water reservoirs was stochastic, with a higher degree of explanation observed in the southern reservoirs compared to the northern reservoirs, divided according to the Qinling-Huai River boundary (Zhang et al., 2024). The assembly processes of eukaryotic plankton in both Danjiangkou Reservoir (DJK) and the Middle Route of the South to North Water Diversion Project (MRP) are predominantly influenced by stochastic processes. However, in comparison to DJK, deterministic processes have a more pronounced impact on MRP, accounting for 39.29% (Zhu et al., 2025). Multivariate statistics and a null model approach dictated deterministic community assembly for both phytoplankton and bacterioplankton across contrasting hydrographic conditions in a subtropical mid-sized river (Jiulong River, southeast China) (Isabwe et al., 2018). These findings indicated that the community assembly mechanisms of plankton were influenced not only by the connectivity and flow regime of water bodies, but also potentially by other ecological and environmental factors.

Phytoplankton was predominantly shaped by deterministic processes from the upstream pristine site toward the downstream urban area along Houxi River in southeast China over the years 2012–2016 (Isabwe et al., 2022). It indicated a balance between dispersal due to fluvial connectivity and local selective environmental heterogeneity. In this study examining the seasonal variations in the assembly of phytoplankton communities in the middle and lower reaches of the Yellow River, we also observed a dynamic equilibrium between species dispersal and environmental heterogeneity. The FV was significantly higher in autumn than in spring, indicating a higher dispersal capacity of phytoplankton in autumn (Figure 7k). Meanwhile, the variability in major water environment parameters, including water turbidity, TOC, pH, SiO_2_, DO, and TP was lower in autumn than in spring (Figure 7). It was found that the homogeneity of the water environment in the middle and lower reaches of the Yellow River, including sediment concentration, is significantly higher in autumn than in spring.

The analysis of the neutral community model and null model revealed that stochastic processes (dispersal limitation) were more prominent in spring, summer, and autumn, while deterministic processes (heterogeneous selection) played a greater role in winter along Jihongtan Reservoir, the terminal storage reservoir of the Yellow River to Qingdao Water Diversion Project (Yang et al., 2025). Deterministic effects were also found to be mainly driven by homogeneous selection influence eukaryotic biofilm microbiota along MRP in spring (Huang et al., 2025). These findings indicate that environmental heterogeneity plays more important roles in phytoplankton community assembly in dry seasons (spring and/or winter), which are characterized by higher environmental gradients. In contrast, spatial factors dominate phytoplankton community assembly during wet seasons (summer and/or autumn), when the environmental gradients are lower.

During autumn, the Yellow River experiences high flow conditions. Strong water flow shear forces cause large-scale riverbed sediment resuspension (Yin et al., 2023; Xu et al., 2022). This leads to significantly increased sediment concentrations. The intense mixing action of high-sediment water flow causes phytoplankton to show random distribution patterns in the water column. It effectively disrupts the spatial stability required for niche differentiation. In contrast, the Yellow River experiences relatively stable hydrological conditions during spring. These stable conditions promote phytoplankton niche differentiation. This pattern aligns with the findings in the entire Yellow River Basin (Yi et al., 2024).

The runoff of the Yellow River shows significant seasonal variation characteristics. Among them, the flow is relatively low in spring and winter, while it remains at a relatively high level in summer and autumn (Yang et al., 2012). It is worth noting that winter, as a typical dry season, has relatively stable hydrological conditions with smaller fluctuations. Based on these hydrological characteristics, we speculate that the construction pattern of phytoplankton communities in the middle and lower reaches of the Yellow River is as follows: in spring and winter, it may be dominated by decisive processes dominated by ecological niches, while in summer and autumn, it may transform into dispersal limitation dominated by diffusion. However, due to the lack of field investigation data in winter, the scientific validity of this inference still requires verification through subsequent systematic seasonal sampling and comprehensive analysis.

### 4.3 Multiple factors influencing phytoplankton composition

Correlation analysis using Bray–Curtis distance revealed that the dissimilarity of phytoplankton communities in both autumn and spring was significantly influenced by environmental factors, geographic separation, and elevation differences (*p* < 0.05, Figure 4). RDA analysis further demonstrated that all three kinds of factors influenced the dominant phytoplankton species in the middle and lower reaches of the Yellow River. This is partially consistent with the findings for the entire Yellow River (Han et al., 2024), which indicate that water quality is a predominant driver of phytoplankton community composition, outweighing the influences of elevation and land use.

Elevation and geographic factors (MEM 1, MEM 2) contributed more to the phytoplankton communities in the middle and lower reaches of the Yellow River in autumn compared to in spring. The difference in elevation between the highest and lowest points in the middle and lower reaches of the Yellow River is 904 m. Consequently, elevation serves as a critical influencing factor in shaping phytoplankton community composition during both seasons. Elevation essentially reflects climatic conditions and certain environmental factors (Zhao et al., 2023). In studies on planktonic algae and Bacillariophyta communities across the Yellow River Basin, the impact of elevation has also been identified (Han et al., 2024). The middle and lower reaches of the Yellow River span a distance of 2,064 kilometers. This extensive geographical range, combined with higher flow velocities and greater variability in the water body during autumn (Figure 7k), have emerged as key factors influencing the phytoplankton community structure in autumn, particularly the dominant species.

Phytoplankton communities were influenced more by environmental factors in spring than in autumn. Two factors including SiO_2_ and pH were screened to be the most important environmental factors in spring. Our study revealed a significant correlation between the concentration of SiO₂ and the community structure of phytoplankton. Specifically, SiO₂ exhibited negative correlations with dominant Cyanophyta and Chlorophyta but was significantly positively correlated with Bacillariophyta (Figure 8b). Bacillariophyta communities require sufficient dissolved silicates (Song et al., 2020). From a physiological perspective, the primary component of Bacillariophyta cell walls is hydrated amorphous silicon dioxide (SiO₂·nH₂O), and its biosynthesis process is highly dependent on dissolved SiO₂ in the environment (Martin-Jézéquel et al., 2000). Additionally, the SiO₂ content in Bacillariophyta cells was found to be significantly positively correlated with their volume (Conley et al., 1989). Studies have demonstrated that during water and sediment regulation in Xiaolangdi Reservoir, an increase in SiO₂ concentration coincides with an increase in phytoplankton biomass (Song et al., 2020), further corroborating the significant correlation between SiO₂ and the dominant species identified in our study.

The relationship between pH and phytoplankton is mutually causal. Existing studies have shown that an increase in phytoplankton biomass leads to an increase in the pH value of water bodies. This is because phytoplankton absorb carbon dioxide through photosynthesis, causing the carbon dioxide balance system to shift towards an increase in pH (Oberlander et al., 2025). pH regulates phytoplankton nutrient absorption by affecting enzyme synthesis and cellular oxidative stress responses. This further influences their growth and metabolic processes (Santini et al., 2022).

One environmental factor, i.e., TN was screened to influence phytoplankton communities in autumn. Further investigation demonstrated that nitrate nitrogen (NO₃-N), which represents the predominant form of inorganic nitrogen in aquatic systems, exhibited significantly lower concentration levels compared to those observed in spring (*p* < 0.01). This seasonal variation in nitrogen forms is likely to exert a substantial influence on phytoplankton communities. When NO₃-N concentrations in water bodies markedly decrease during autumn, phytoplankton may enhance their competitive uptake of ammonium (NH_3_-N) (Wan et al., 2018). While ammonia nitrogen (NH_3_-N) is generally considered as the preferred nitrogen source for phytoplankton, prior studies have confirmed that diverse phytoplankton groups, such as Bacillariophyta and Chlorophyta, possess nitrate reductase systems and are capable of utilizing nitrate nitrogen for metabolic processes (Eppley et al., 1969; Huang et al., 2024).

Although this study did not directly detect the significant impact of sediment on phytoplankton communities, it is worth noting that during the period with higher sediment content (autumn), the concentrations of key nutrients such as TN and TP in the water body showed a downward trend. This phenomenon may be related to the special physicochemical processes of water bodies with high sediment content. A large number of suspended sediment particles fix the nutrients in the aqueous phase through adsorption, forming granular bound nutrients, thereby reducing their bioavailability capacity (Wu et al., 2025; Jin et al., 2005). Meanwhile, the increase in turbidity caused by sediment will significantly weaken the light transmittance of water bodies, inhibit the photosynthetic efficiency of phytoplankton, and thereby affect their absorption and assimilation capacity of dissolved nutrients (Wang et al., 2016). Since this study only explored the seasonal similarities and differences in the construction mechanism of phytoplankton communities in the middle and lower reaches of the Yellow River, subsequent studies should delve deeper into the influence mechanism of sediment on phytoplankton.

## 5 Conclusion

A total of 110 phytoplankton species, belonging to 7 phyla and 69 genera, were identified in the middle and lower reaches of the Yellow River within the high sand content section during this survey. Among these, the Chlorophyta phylum was dominant in terms of species richness, while the Cyanophyta phylum dominated cell density. NMDS analysis based on BC distance revealed significant differences in community structure between spring and autumn, with α-diversity evenness being significantly higher in spring than in autumn. β-diversity decomposition results indicated that turnover groups predominated in both seasons, with community differences primarily arising from nestedness groups. Calculations of the DNCI and the MST revealed that community assembly was primarily influenced by dispersal constraints and stochastic processes in autumn, whereas ecological niche effects and deterministic processes were more prominent in spring. Additionally, geographic, elevation, and environmental factors shaped community patterns to varying degrees through diffusion limitations and environmental filtering, with the most significant environmental factors being TN in autumn and SiO_2_ and pH in spring. Overall, the assembly of phytoplankton communities in the highly sandy region of the middle and lower reaches of the Yellow River occurred under the combined influence of diffusion limitations and ecological niche effects, which played varying roles across different seasons. These processes intertwined during ecological succession in response to changing environmental conditions, jointly governing the formation and evolution of community structure.

## Data Availability

The original contributions presented in the study are included in the article/Supplementary material, further inquiries can be directed to the corresponding authors.
