# Supplementary material for: Seasonal reversal in phytoplankton assembly mechanisms: stochastic dominance in autumn vs. deterministic control in spring within the middle and lower reaches of the Yellow River
Source: Front Microbiol. 2025 Jul 14;16:1610438. doi: 10.3389/fmicb.2025.1610438 (PMC12301358; doi:10.3389/fmicb.2025.1610438)
Supplement: Supplementary file 1 [file Data_Sheet_1.docx]

**Seasonal Reversal in Phytoplankton Assembly Mechanisms: Stochastic Dominance in Autumn vs. Deterministic Control in Spring within the middle and lower reaches of the Yellow River**

Dahai Zeng^1,2,3^, Houkuan Ding^1,2,3^, Yuanyuan Tang^1,2,3^, Yunni Gao^1,2,3*^, Jialin Jin^1,2,3^, Xiaofei Gao^1,2,3^, Jingxiao Zhang^1,2,3^, Huatao Yuan^1,2,3^, Jing Dong^1,2,3*^, Xuejun Li ^1,2,3*^

1: College of Fisheries, Henan Normal University, Xinxiang 453007, P.R.China

2: Observation and Research Station on Water Ecosystem in Danjiangkou Reservoir of Henan Province, Nanyang, 474450, P.R.China

3. The National Ecological Quality Comprehensive Monitoring Station (Hebi Station), Hebi 458000, P.R. China

*Corresponding author address: College of Fisheries, Henan Normal University, Xinxiang 453007, Henan Province, China

E-mail address: gaoyn@htu.cn

Supporting Information consists of 5 pages, including this one. There are two Figures, two Tables.


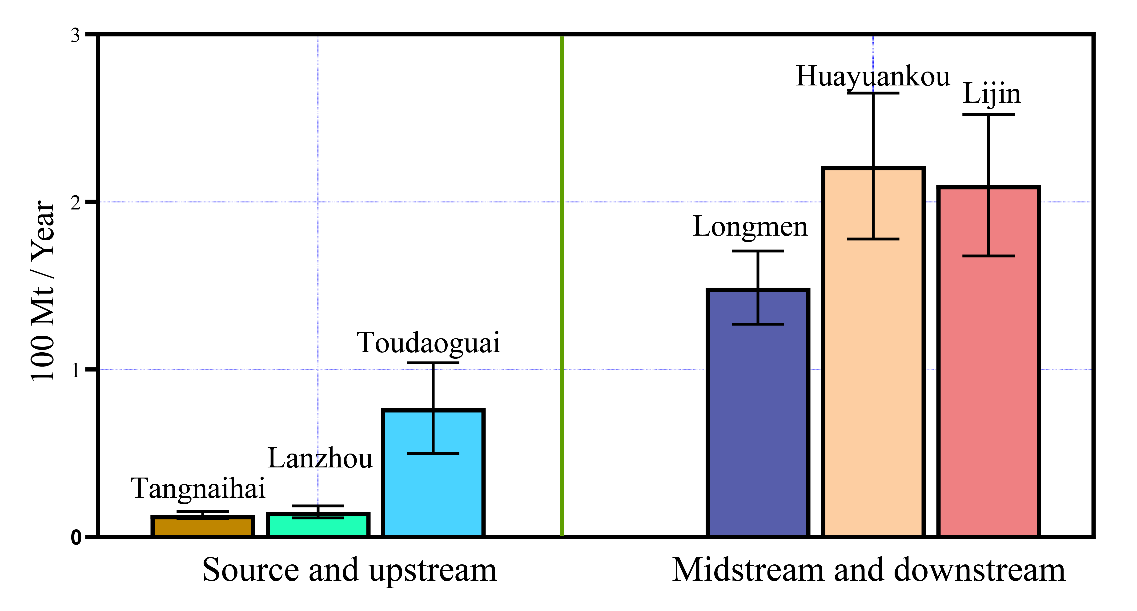


Fig. S1. Annual sediment transport of key hydrological stations of the main stream of the Yellow River from 2019 to 2023

Table S1 Location of sampling sites in the middle and lower reaches of the Yellow River.

| Site | Location | Water Body Type | | | Longitude | Latitude | Region |  |
| --- | --- | --- | --- | --- | --- | --- | --- | --- |
|  |  |  |  |  |  |  |  |  |
| M1* | Wanjiazhai | | Main stream | 111.4269025 | | 39.5673177 | Midstream |  |
| M2* | Fugu | | Main stream | 110.0535142 | | 39.023769 | Midstream |  |
| M3 | BaiShuPing | | Main stream | 110.6764764 | | 37.4412982 | Midstream |  |
| M4 | Wuding River Confluence | | Main stream | 110.425211 | | 37.0426772 | Midstream |  |
| M5 | Qingjian River Confluence | | Main stream | 110.3987941 | | 36.6960623 | Midstream |  |
| M6 | Guxian Dam | | Main stream | 110.4848141 | | 36.3980932 | Midstream |  |
| M7* | Hukou Waterfall | | Main stream | 110.4751692 | | 36.0547563 | Midstream |  |
| M8* | Longmen | | Main stream | 110.5967275 | | 35.6595275 | Midstream |  |
| M9 | Miaoqian Village | | Fen River | 110.4867253 | | 35.3536608 | Midstream |  |
| M10 | Downstream of Fen River Confluence | | Main stream | 110.3713412 | | 35.135239 | Midstream |  |
| M11* | Tongguan suspension bridge | | Wei river | 110.2373425 | | 34.6143897 | Midstream |  |
| M12 | Fenglingdu Bridge | | Main stream | 110.3251576 | | 34.6135552 | Midstream |  |
| M13* | Sanmenxia Reservoir | | Reservoir | 111.1289084 | | 34.7969932 | Midstream |  |
| M14 | Nanshan | | Reservoir | 112.0235414 | | 35.0363741 | Midstream |  |
| M15 | Dahengling | | Reservoir | 112.2709821 | | 34.94788 | Midstream |  |
| M16* | Xiaolangdi Reservoir | | Reservoir | 112.4003089 | | 34.921076 | Midstream |  |
| M17* | Baihe ferry | | Main stream | 112.5393939 | | 34.8762767 | Midstream |  |
| M18 | Yiluo River Estuary | | Main stream | 113.0570509 | | 34.836295 | Downstream |  |
| M19 | Tributary of Yiluo River | | Yiluo River | 113.057821 | | 34.836792 | Downstream |  |
| M20 | Downstream of Yiluo River Confluence | | Main stream | 113.1965632 | | 34.856796 | Downstream |  |
| D1* | Qin River Confluence | | Qin River | 113.2645218 | | 35.0664448 | Downstream |  |
| D2* | Huayuankou | | Main stream | 113.700891 | | 34.9139547 | Downstream |  |
| D3* | Jiahetan | | Main stream | 114.57808 | | 34.926758 | Downstream |  |
| D4* | Gaocun | | Main stream | 115.069155 | | 35.40929 | Downstream |  |
| D5* | Sunkou | | Main stream | 115.904235 | | 35.934123 | Downstream |  |
| D6 | ZhangQiu | | Jindi river | 115.924223 | | 36.028636 | Downstream |  |
| D7* | Aishan | | Main stream | 116.302191 | | 36.269947 | Downstream |  |
| D8* | Luokou | | Main stream | 116.988384 | | 36.727829 | Downstream |  |
| D9* | Lijin | | Main stream | 118.304479 | | 37.521714 | Downstream |  |
| D10 | Kenli | | Main stream | 118.530989 | | 37.604053 | Downstream |  |

*Represents hydrological station, with tributary sampling points located within 3 km of the main stream.

Table S2 Dominance (Y) of dominant phytoplankton species in the middle and lower reaches of the Yellow River.

| Phylum | Species | Dominance index | |
| --- | --- | --- | --- |
|  |  | Autumn | Spring |
| Bacillariophyta | *Melosira varians* | 0.29 | - |
|  | *Fragilaria* sp. | 0.14 | 0.34 |
|  | *Aulacoseira granulate* | 0.05 | 0.06 |
|  | *Cyclotella* sp. | 10.25 | 0.75 |
|  | *Ophiocytium* sp. | 0.11 | - |
|  | *Navicula* sp. | 0.14 | 0.06 |
|  | *Diatoma* sp. | - | 0.11 |
|  | *Asterionella formosa* | - | 0.11 |
| Cyanobacteria | *Pseudanabaena* sp. | 1.82 | 2.32 |
|  | *Microcystis* sp. | 1.59 | 0.17 |
| Chlorophyta | *Scenedesmus bijuga* | 0.11 | 0.29 |
|  | *Elakatothrix* sp. | 0.07 | - |
|  | *Monoraphidium mirabile* | - | 0.34 |
|  | *Scenedesmus quadricauda* | - | 0.09 |
|  | *Monoraphidium contortum* | - | 0.2 |
| Euglenophyta | *Trachelomonas* sp. | 0.11 | - |

*The calculation formula is: $Y=f_{i}\times n_{i}/N$. In the formula, $n_{i}$represents the quantity of the i-th species; N is the total quantity of all species in the community; and $f_{i}$ is the frequency of occurrence of the species across various sample points. Species with Y ≥ 0.02 are identified as dominant species.
